# Broadband infrared light source by simultaneous parametric down-conversion

**DOI:** 10.1038/s41598-021-97531-w

**Published:** 2021-09-09

**Authors:** Masayuki Hojo, Koichiro Tanaka

**Affiliations:** Department of Physics, Science, Kyoto University, Kitashirakawa-Oiwake, Sakyo, Kyoto, 606-8224 Japan

**Keywords:** Lasers, LEDs and light sources, Quantum optics

## Abstract

Spontaneous parametric down-conversion is an essential tool for a quantum light source in the infrared region ranging 2–5 µm for the purpose of material identification, chemical analysis, and gas sensing. So far, photon pairs from the process in a nonlinear crystal have low tunability and a narrow spectral range because of the phase-matching condition. Here, we propose a novel type of spontaneous parametric down-conversion processes that overcomes these challenges, where two photon pairs are simultaneously produced in the visible and infrared regions in periodically poled stoichiometric lithium tantalite. It allows broadband and tunable generation of infrared photon pairs that can be employed as an alternative light source for quantum infrared spectroscopy.

## Introduction

Quantum infrared spectroscopy^1–4^ (QIS) is an emerging method for investigating the fundamental absorption band and characterizing the properties of materials. This technique employs a spontaneous parametric down-conversion (SPDC) process, where one photon (pump) is split into two entangled photons (signal and idler) in a nonlinear crystal. In QIS, two SPDC processes, which occur in two identical nonlinear crystals, quantumly interfere each other. One notable feature is that the two signals show an interferogram when the optical phase or amplitude vary not only between the two signals but even between the two idlers^5^. Therefore, using the SPDC photon pairs whose wavelengths are in the visible and IR region, respectively, the spectral information about the IR idlers can be obtained by measuring the visible signal interferogram.

The current issue in the QIS is that the SPDC light source has a limitation to its performance. Broadband and tunable generation is a key element for analyzing samples over a wide spectral range. The spectral window and tunability of the SPDC light source have so far been limited by the phase-matching condition, because the frequencies of the signal-idler pairs are determined by the pump wavelength, the radiation angles of the SPDC photons, and the refractive index of the nonlinear crystal.

A number of methods have been devised to complement the spectral coverage of the IR idlers. Using a short crystal in a non-collinear process^1,6,7^ or using a chirped poled crystal^6,8,9^ is a direct method to broaden the emitting range, but it comes at the cost of poor efficiency. Furthermore, employing a large radiation angle (with a conventional angle more than 10°) requires a complicated optical system. Another method, group-velocity matching^10,11^, improves the spectral range in the collinear condition, though it suffers from limited selectivity of emitting wavelengths. Thus, the limitations of these techniques reduce the brightness and flexibility of the SPDC emitter and complicate the experimental layout. It would be a significant advance to overcome the tradeoff between the broadness (or tunability) and simplicity (or brightness) of the SPDC emitter.

**Figure 1:**
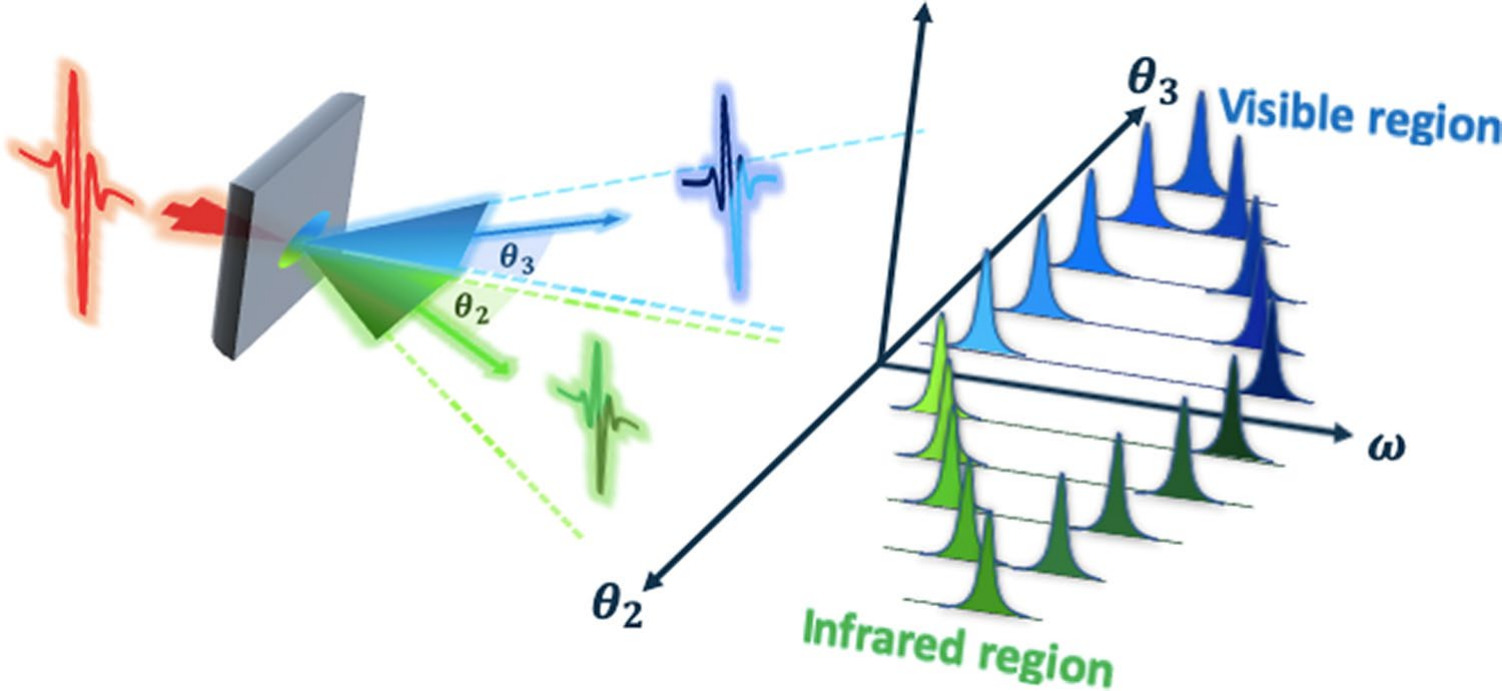
Conceptual diagram of two simultaneous SPDC processes. From the incident pump light, two SPDC pairs are simultaneously produced under the same QPM condition, in which signals are generated in the visible region and idlers in the IR region. The relation between the SPDC idler-signal wavelengths and their output angle ${\theta }_{2}, {\theta }_{3}$ under the non-collinear process indicates that the broadband IR range can be covered by the idlers emitted within a given angle.

In this report, we show an alternative generation scheme of a SPDC process which balances the above tradeoff. Two simultaneous SPDC processes in a given crystal enable ultrabroad and tunable IR idlers to be generated by using a quasi-phase-matched crystal in which the phase mismatch is restricted by the periodic construction. Figure 1 shows a conceptual diagram for the proposed scheme. By adjusting the pump wavelength in a given range, two pairs of visible signal and IR idler photons are generated with the pump light in the collinear geometry. As the angle *θ*_2_ (*θ*_3_) with respect to the pump light is increased, the wavelengths of the two idlers (signals) come close to each other. This process offers a broad and tunable emitting range with a smaller output angle than that of the conventional single SPDC process. Moreover, we also introduce a theoretical description of the spectrum and total brightness of the SPDC. Our experimental results are well explained by this theory.

## Results

### Simultaneous parametric down conversion process

**Figure 2:**
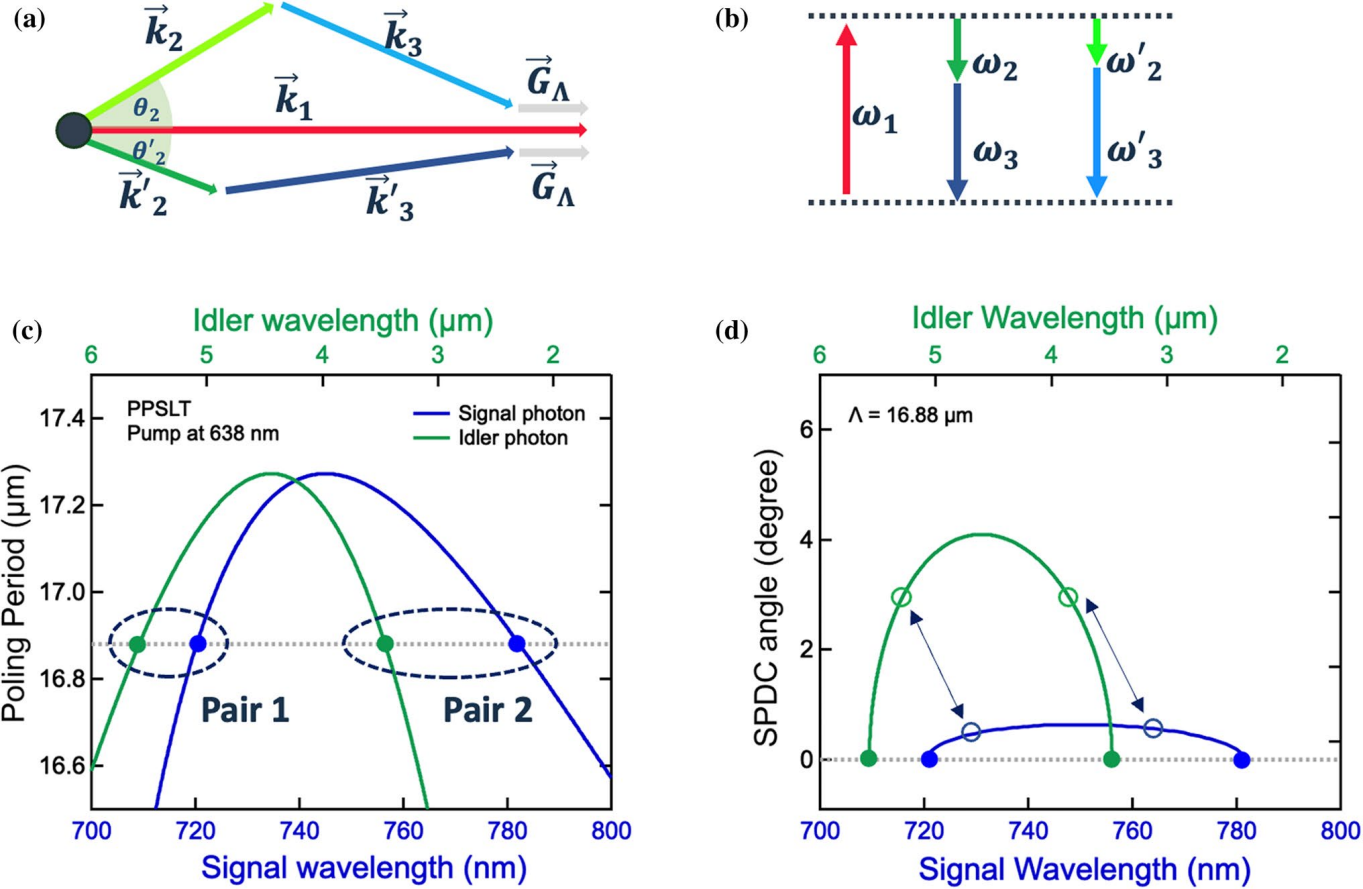
Schematic diagrams and theoretical simulations of quasi-phase-matching condition. (**a**) Conservation law for wave vectors in SPDC. Two signal-idler pairs phase-matched for the same pump and $\overrightarrow{{G}_{\Lambda }}$. The poling superlattice vector $\overrightarrow{{G}_{\Lambda }}$ is assumed to be parallel to the pump wave vector. (**b**) Energy diagram of related photons for SPDC in the crystal. (**c**) Relation between the poling period of the crystal and SPDC wavelengths for the pump at 638 nm. The bottom axis represents the SPDC signals (blue curve) and the top the SPDC idlers (green curve). With the periodicity of 16.88 µm, two pairs (Pair 1: 720 nm and 5.6 µm. Pair 2: 780 nm and 3.5 µm) are simultaneously quasi-phase-matched in the same condition. (**d**) Dependency of the emission angle of the SPDC signals and idlers on their wavelengths. The vertical axis represents the SPDC output angle. The dashed circles indicate the collinear process (**c**) and double arrows indicate the non-collinear process in the case of the idler angle of 3°.

The brightness of the SPDC process depends on the quasi-phase mismatch in periodic crystals as follows:

1 $$\Delta k = \left| {\overrightarrow {{k_{1} }} - \overrightarrow {{k_{2} }} - \overrightarrow {{k_{3} }} - \overrightarrow {{G_{\Lambda } }} } \right|,$$

where $\left|\overrightarrow{{k}_{i}}\right|=2\pi n\left({\lambda }_{i},T\right)/{\lambda }_{i}$ denotes the wave vector of the pump ($i$ = 1), idler ($i$ = 2) and signal ($i$ = 3) light. $n\left({\lambda }_{i},T\right)$ is the refractive index of the crystal at temperature $T$ and wavelength ${\lambda }_{i}$. $\left|\overrightarrow{{G}_{\Lambda }}\right|=2\pi /\Lambda$ is the superlattice vector obtained from the periodicity Λ for compensating the phase mismatch. Figure 2a,b show the QPM conditions, $\Delta k=0$ and the energy conservation, where two SPDC processes are simultaneously allowed for the identical pump laser and periodicity of the crystal. We found that these conditions are satisfied only for limited pump wavelengths in periodically poled stoichiometric lithium tantalite (PPSLT) and periodically poled lithium niobate (PPLN).

Figure 2c shows the signal and idler wavelengths in the collinear configuration for $\Delta k=0$ in Eq. (1) for various periodicities Λ in PPSLT calculated using the temperature-dependent Sellmeier equation^12^. We chose a pump wavelength of 638 nm and T = 25 °C, which was also employed in our experiment as explained below. In the case of a periodicity of 16.88 µm (the dashed line), two pairs are emitted in which ${\lambda }_{2}$ of pair 1 is 3.5 µm and ${\lambda }_{3}$ of pair 1 is 780 nm and those of pair 2 are 5.6 µm and 720 nm. One can determine that two such processes are only allowed for periodicities less than 17.3 µm. Furthermore, the gap between the wavelengths of the signals or idlers gets smaller as the periodicity changes. This enables broadband generation (3.5–5.6 µm) using a chirped grating crystal, in which the periodicity varies from 16.8 to 17.3 µm in the direction parallel to the grating vector.

The non-collinear QPM configuration with a period of 16.88 µm was also calculated, as shown in Fig. 2d. The blue and green dots on the dashed line indicate the collinear pairs (${\theta }_{2}={\theta }_{3}=0$), and the double arrows indicate the non-collinear pairs (${\theta }_{2}=3$°). In this process, the visible range and the IR range between the collinear QPM pairs can be covered with output angles up to 0.7° for the signals and 4.1° for the idlers. It follows that this non-collinear process allows a broad 3.5–5.6 µm spectral window wherein all photons are gathered within an output angle of 4.1°. Note that the emitting angles of the pair should be different.

**Figure 3:**
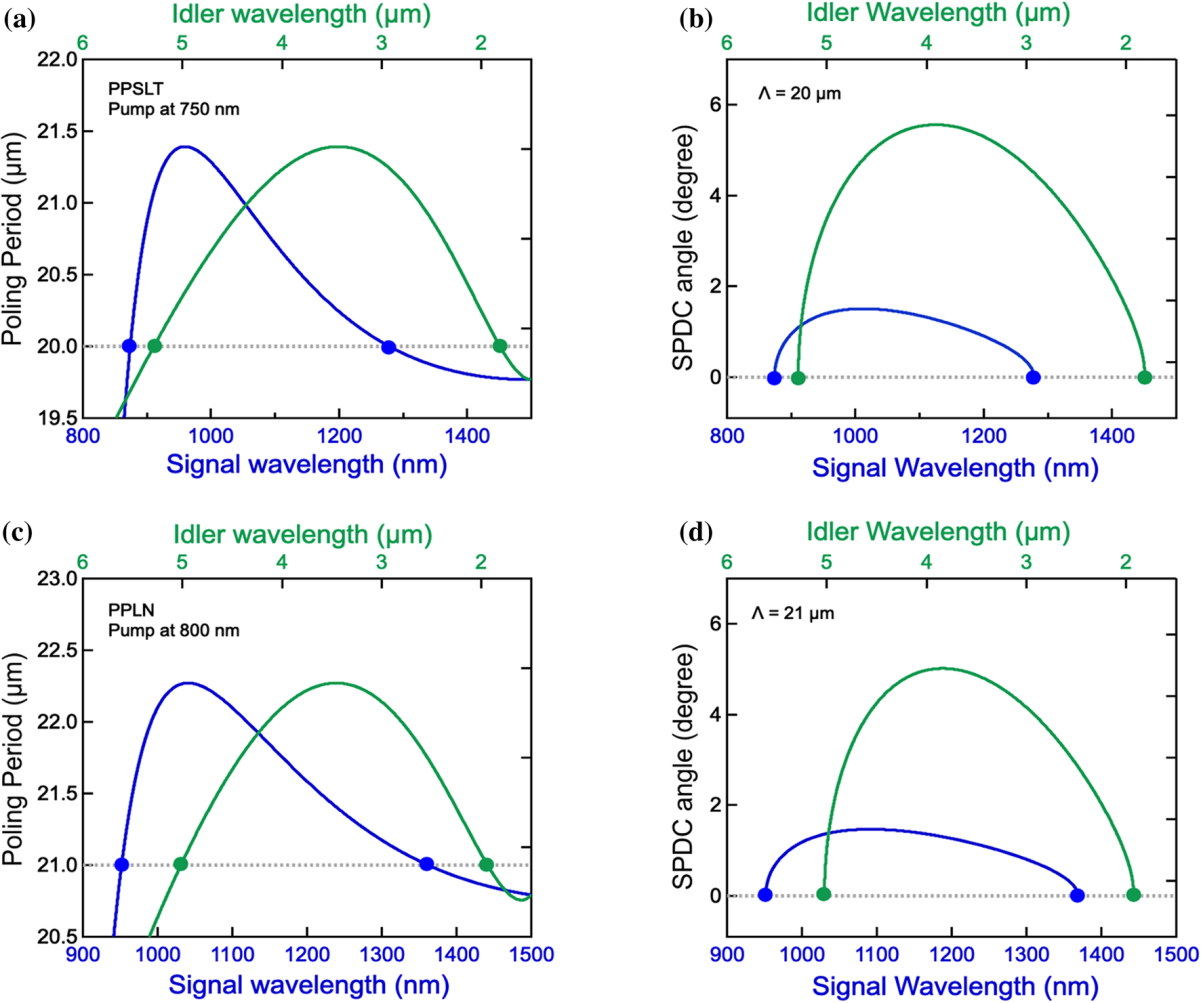
Theoretical calculations of the optimized QPM conditions for simultaneous SPDC in PPSLT (**a**,**b**) and PPLN (**c**,**d**). Polling periods in the collinear process are represented by the vertical axes for the optimized conditions in PPSLT (**a**) and in PPLN (**c**), where pump wavelengths are set to 750 nm and 800 nm, respectively. In (**a**), with the periodicity of 20 µm, two pairs (Pair 1: 880 nm and 5.2 µm. Pair 2: 1280 nm and 1.8 µm) are simultaneously quasi-phase-matched in the same condition. In (**c**), with the periodicity of 21 µm, two pairs (Pair 1: 950 nm and 5 µm. Pair 2: 1360 nm and 2 µm) are simultaneously quasi-phase-matched in the same condition. Figure (**b**), (**d**) show the corresponding non-collinear QPM curves. The vertical axes represent the output angles of the SPDC photons. In (**b**), for the pump at 750 nm, all photons within output angles of 5.7° are produced in the range of 1.8–5.2 µm. In (**d**), for the pump at 800 nm, all photons within output angles of 5.2° are produced in the range of 2–5 µm.

Furthermore, we calculated the QPM conditions for various pump wavelengths and found that simultaneous SPDC processes occur with the pump wavelength in the range of 600–900 nm. We have an optimized condition for the idler bandwidth limited by the PPSLT absorption edge (5.5 µm) when the pump is set around 750–780 nm. The details of the calculation procedure are in Supplementary Information 1. Figure 3a,b show the QPM curves for the pump at 750 nm in PPSLT. With the period 20 µm, the simultaneous pairs (880 nm and 5.2 µm, and 1280 nm and 1.8 µm) are phase-matched in the collinear process. Using the non-collinear process, the SPDC idlers are emitted at 1.8–5.2 µm with the output angle 5.6°. It is a significant feature that broadening the emitting range does not require increasing the output angle so much, which means that over 100 THz spectral window can be obtained in the near-collinear configuration.

We also investigated the case of PPLN, which has a similar dispersion with PPSLT. We found that the simultaneous processes could be occurred also in PPLN. The optimized pump is at about 0.8 µm as shown in Fig. 3c,d. The 2–5 µm bandwidth of SPDC idlers can be also obtained in the near-collinear process, the specifics of which are shown in Supplementary Information 2.

We also calculated the frequency and angular dependence of the power of the non-collinear SPDC signals. The signal power per unit frequency $d{\omega }_{3}$ and transverse wavevector $d{{\varvec{K}}}_{3}$ is described as^13^

2 $$dP_{3} = \frac{{\hbar \omega_{3}^{2} \omega_{2} L^{2} d^{2} P_{1} }}{{4\pi^{3} c^{3} \varepsilon_{0} n_{1} n_{2} n_{3} }}{\text{Sinc}}^{2} \left[ {\frac{\Delta kL}{2}} \right]d\omega_{3} d{\varvec{K}}_{3} ,$$

where $d$ is the effective nonlinear coefficient, $L$ is the crystal length, and ${P}_{1}$ is the pump power. Sinc $\left[x\right]$ ≡ sin $(x)/x$ reflects on the phase mismatch. In cylindrical coordinates, $d{{\varvec{K}}}_{3}$ is expressed as

$$d{{\varvec{K}}}_{3}={k}_{r,3}d{k}_{r,3}d{\varphi }_{3},$$

where ${k}_{r,3}=\sqrt{{k}_{x,3}^{2}+{k}_{y,3}^{2}}$. Here, by using the angular symmetry around the $z$-axis, we have $\int d{\varphi }_{3}=2\pi$. We introduce the signal emitting angle ${\theta }_{3}$ where $\mathrm{tan}{\theta }_{3}={k}_{r,3}/{k}_{z,3}$. Thus, Eq. (2) becomes

3 $$dP_{3} = \frac{{\hbar \omega_{3}^{4} \omega_{2} n_{3}^{2} L^{2} d^{2} P_{1} }}{{2\pi^{2} c^{5} \varepsilon_{0} n_{1} n_{2} }}\frac{{\sin \theta_{3} }}{{\cos^{3} \theta_{3} }}{\text{ Sinc}}^{2} \left[ {\frac{\Delta kL}{2}} \right]d\omega_{3} d\theta_{3} .$$

Equation (3) is nothing but the differential power radiated at the frequency range of ${\omega }_{3}\sim {\omega }_{3}+d{\omega }_{3}$ and within the emitting range ${\theta }_{3}\sim {\theta }_{3}+d{\theta }_{3}$. By integrating over ${\theta }_{3}$, we obtain the spectra of the non-collinear SPDC signals, and by integrating over ${\omega }_{3}$, we obtain the total brightness of the whole process. This allows us to predict the SPDC spectral curve and conversion efficiency. Note that the total brightness is proportional to the crystal length $L$, which is consistent with the conventional SPDC^14^, the details of which are explained in Supplementary Information 3.

### Experimental setup

**Figure 4:**
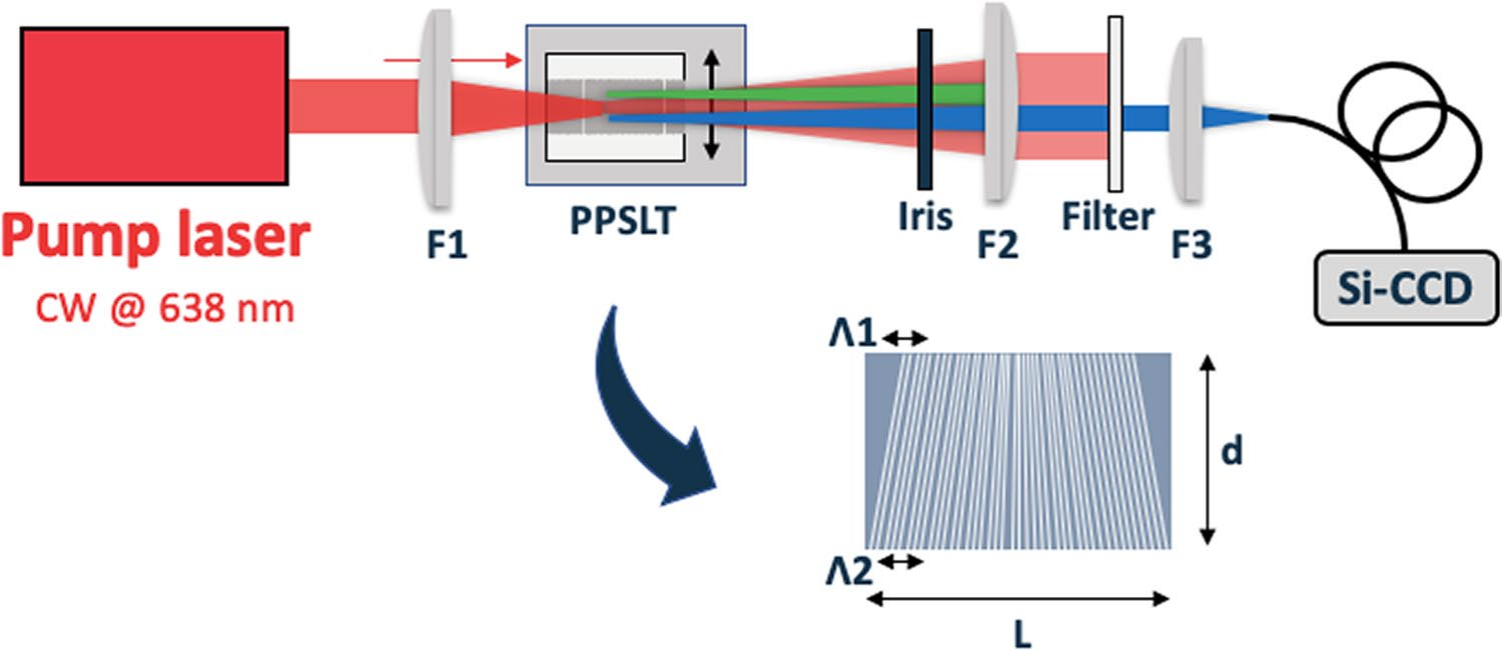
Schematic layout of the experimental setup. F1: 100 mm lens for focusing the pump into the crystal. Iris: Movable aperture with a variable radius from 0.5 mm to 5 mm. F2: 200 mm lens for collimating the output signal. Filter: Bandpass filter with 700–900 nm passing range. F3: 50 mm lens for coupling the signal with the detector.

The experimental setup for broadband IR light generation is illustrated in Fig. 4. Here we chose 638 nm (THORLABS, L638P200, 50 mW) as the pump wavelength, which corresponds to the case of Fig. 2c,d. Note that we used the pump at 638 nm for demonstrating the simultaneous processes using a sensitive Si detector, in which all SPDC signals were expected to be produced in the visible region.

**Figure 5:**
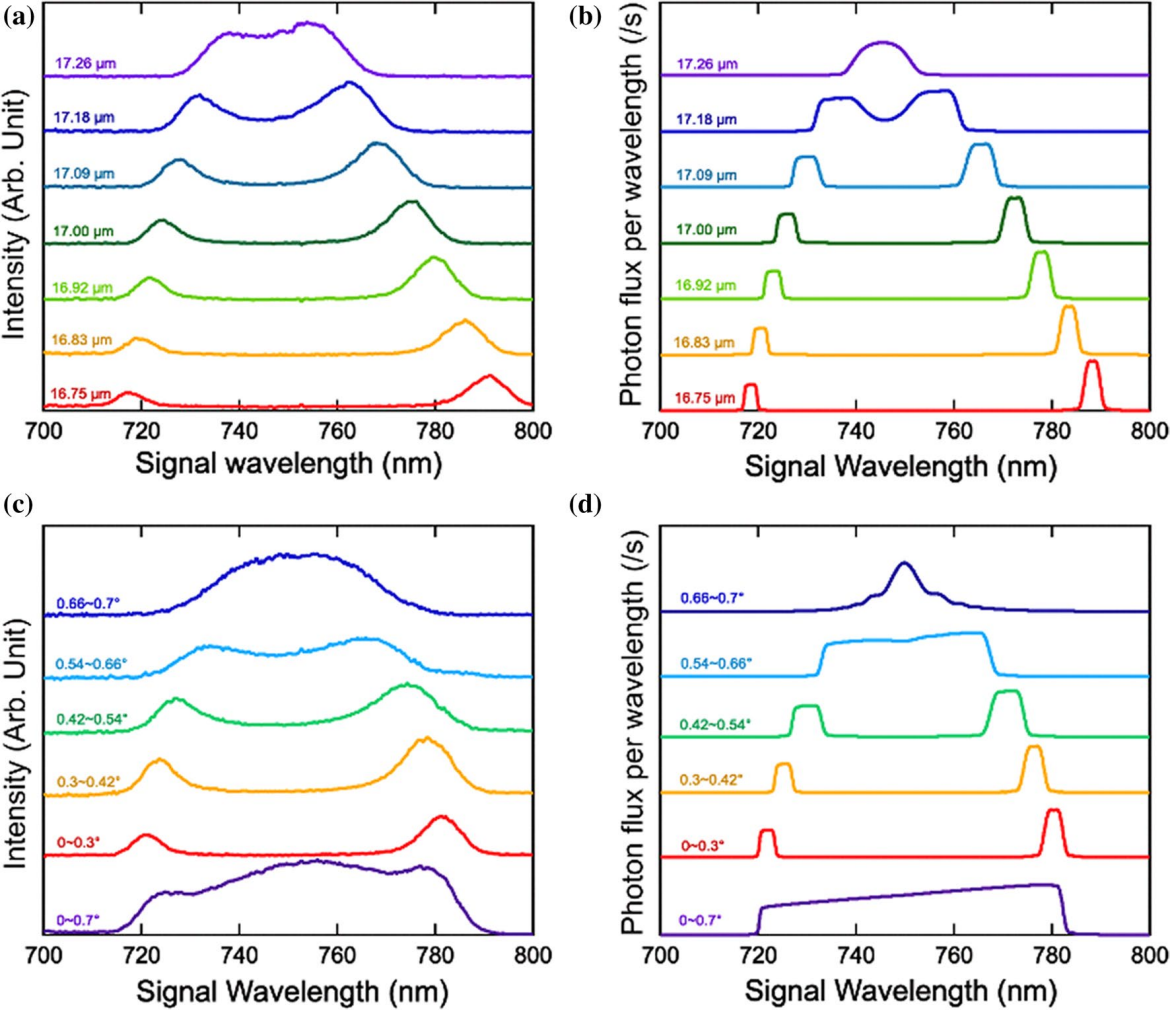
(**a**) SPDC signal spectra for Fanout-PPSLT with several periodicities in the collinear geometry. The two peaks correspond to phase-matched wavelengths. (**b**) Simulated collinear spectra for the periodicities measured in (**a**). (**c**) SPDC signal spectra for different emitted angles with a periodicity of 16.88 µm. The bottom broadband spectrum (0°–0.7°) includes all non-collinear components. (**d**) Simulated non-collinear spectra for the angles measured in (**c**).

We employed type-0 PPSLT (Oxide Corp., L = 11 mm, d = 5 mm) with a fan-out structure as the nonlinear crystal, where the period could be varied from 16.6 µm (${\Lambda1 }$) to 18.3 µm (${\Lambda2}$) by sliding the crystal at 90° to the beam direction. The crystal was maintained at 25 °C and mounted on a movable stage to allow full probing of the period range with an accuracy of 10 nm. The pump beam was focused into the crystal by using a 100 mm lens (F1) with a waist radius of ~ 100 µm. The pump wave vector and the QPM grating vector of the crystal were collinear along the crystal. After the crystal, an iris was set to restrict the radiation angle of the non-collinear SPDC signal. The optics for visible light blocked the idlers and only the signal was collimated by a 200 mm lens (F2) and focused into a fiber coupled Si-CCD array (Ocean Optics) by a 50 mm lens (F3). The resolution $\Delta \lambda$ of this system was limited by the detector (7 nm) and the geometric arrangement. The photons produced at a given angle in the frontside and backside in the crystal make the angle resolution lower, in which the lower the resolution is obtained for the larger the angle. The quantitative estimation of the angle resolution will be given with the results of Fig. 5d. An interference filter mostly rejected the pump. We performed two types of experiment: measurement of the periodicity dependence in the collinear method and measurement of the emitting angle dependence.

### Simultaneous parametric down conversion in PPSLT

To investigate the collinear condition, signals were propagated at an angle within 0.3° by narrowing the radius of the iris. The spectra were obtained for each period by sliding the crystal at a constant rate, which are shown in Fig. 5a. The red curve is the case of 16.75 µm. The spectrum is composed of two peaks, the center wavelengths of which correspond to those of the collinear phase-matched signals. This is a non-trivial feature wherein the intensity of the shorter one was weaker than that of the longer. It can be explained by the inclination of the phase-matching curve in Fig. 2c, where the steeper the curve is, the more rapidly $\Delta k$ changes around $\Delta k=0$. According to Eq. (3), a finite power can be produced only where $\Delta k$ is around 0. Therefore, the two peaks had different intensities in the same aperture.

Next, we investigated the period dependence of this process. By changing the periodicity from 16.75 to 17.26 µm, the two peaks converge together. The wavelengths of the peaks in Fig. 5a are those of the SPDC signals in the collinear QPM condition and are qualitatively reproduced by the theoretical calculation of Eq. (3), as shown in Fig. 5b. The calculation integrated over the signal angle ${\theta }_{3}$ from 0° to 0.3° in Eq. (3). The simulated curves also reproduce the difference in the intensity between the two peaks.

Finally, we measured two spectra of signals within ${\theta }_{3}+\Delta \theta$ and within ${\theta }_{3}$, subtracting the second data from the first, and obtained the non-collinear spectra restricted to the angle range ${\theta }_{3}\sim {\theta }_{3}+\Delta \theta$ (corresponding to the signals in the ring area). Here we experimentally defined ${\theta }_{3}$ as the angle of the range restricted by the iris against the center of the crystal. Figure 5c shows the non-collinear spectra in several angle ranges with a periodicity of 16.88 µm. The bottom purple curve reflects the spectrum of all photons within 0.7°. One can see broadband generation covering 720–780 nm. The corresponding IR idlers should also have a broad spectral window covering a wide range from 3.5 to 5.6 µm within 4.1°. The second curve from the bottom is within 0°–0.3°, corresponding to the collinear process. When changing the restricted angle, a peak shift similar to the one in Fig. 5a was observed. We also calculated the angular dependence of the signal spectra by using Eq. (3), as shown in Fig. 5d. The theoretical simulations qualitatively show good agreement with the measured spectra.

One can see that the experimental spectra are broadened more widely than the theoretical ones. The broadening is attributed mainly to two factors. The first one is the systematic resolution. In the collinear process, the detector resolution determines the shapes of the spectra. However, the deviation due to the long crystal could not be neglected for the non-collinear photons. Although 11 mm thickness of the crystal equals to, which corresponds to 1 nm deviation according to the QPM curve of Fig. 2d, the angle deviation of 0.05° is occurred at 0.7°, corresponds to 26 nm deviation. Next, the second factor is that the deviations of the QPM condition were reflected on broadening. Increasing the detecting angle, the QPM curve in Fig. 2d comes shallower, which means that the signals with more different energies are included in larger angle ranges. Therefore, the effect of broadening is especially larger in the non-collinear spectra.

In summary, the two experiments and theoretical analyses demonstrated the simultaneous SPDC process and the features of the SPDC signals. A similar work reported the SPDC idler bandwidth of 2.9–4.7 µm^11^, in which a short crystal were employed for broadening in the collinear process by restricting the phase-mismatch $\Delta kL$. However, we can obtain the broadband spectra using a long crystal. This is because the simultaneous condition allows the broad phase-matching bandwidth in small angle ranges. Therefore, it means that we may obtain not only broadband but also highly efficient SPDC photons. The experimental estimation of brightness of the SPDC photon will be discussed in the next section.

It is noteworthy that we used the pump at 638 nm, which was not in the optimized condition as explained in Fig. 2c,d. In the case of the pump at 750 nm, the range 880–1280 nm of the signals and the range 1.8–5.2 µm of the idlers are expected to be obtained in the same process.

### Total flux of signal photons

We tested the possibility of compatibility between total brightness and the bandwidth of the SPDC signals. The total flux of the non-collinear process was estimated by summing the photons detected in all of the CCD pixels. We evaluated the photon flux of the signal by integrating the counts in the range 720–780 nm with a detector sensitivity of 80 photons/count. For a pump with an average power of 50 mW, the total flux was estimated to be $1.4\times {10}^{10}$ photon/s. This value is comparable to those of previous reports in which a waveguide was used to enhance the brightness^15^. Moreover, our method can employ the near-collinear configuration, so that it is possible to use the crystal more than twice as long as the reported non-collinear broadband SPDC^1,6,7^. This means that process efficiency can be obtained more than twice as high in this process because, as described above, the total flux is proportional to the crystal length.

We also verified Eq. (3) in terms of the SPDC brightness. The total flux ${\Phi }_{3}$ integrated over all emission angle and frequency is

4 $${\Phi }_{3} = \int {\frac{{dP_{3} }}{{\hbar \omega_{3} }}} = \iint {d\omega_{3} d\theta_{3} }\frac{{\omega_{3}^{3} \omega_{2} n_{3}^{2} L^{2} d^{2} P_{1} }}{{2\pi^{2} c^{5} \varepsilon_{0} n_{1} n_{2} }}\frac{{\sin \theta_{3} }}{{\cos^{3} \theta_{3} }}{\text{ Sinc}}^{2} \left[ {\frac{\Delta kL}{2}} \right].$$

Here, we assumed an effective nonlinear coefficient d of 15.1 pm/V^16^ and a crystal length L of 11 mm. The resulting theoretical total brightness is $5.82\times {10}^{11}$ photon/s. The gap of an order of magnitude between the experimental and theoretical value can be ascribed to the reflection loss ${a}_{1}(\sim 0.7)$ of the pump and signal at the boundary of the crystal, to the optical losses ${a}_{2}(\sim 0.7)$ of the fiber coupling and the pump-cut filter, and to the process loss ${a}_{3}(\sim 0.4)$ of the quasi-phase-matched crystal^17^.

## Conclusion

In conclusion, we proposed and demonstrated a novel SPDC scheme that allows broadband and tunable generation of SPDC photon pairs in the IR and visible region by using a periodically poled nonlinear crystal. In the case of 638 nm pump light, by changing the periodicity from 16.8 to 17.3 µm or employing a radiation angle of 0°–4.1°, the wavelengths of idler photons covered a range of 3.5–5.6 µm. Moreover, the high total flux of the SPDC signal characterized our method as highly efficient. This technique will bridge the gap between the demands and technical limitations of broadband and tunable radiation.

It is a pressing issue that IR idler photons were not directly observed in this experiment. However, the photon number of $1.4\times {10}^{10}$ photon/s corresponds to ${10}^{-14}$ W/nm for the IR idlers, which cannot be detected by a semiconductor detector, but might be able to be detected with an up-conversion IR detector^18^ or superconductive-single photon counter (SSPD)^19^.

Furthermore, our method is expected to be not only for the broadband infrared source but also for the quantum entangled photon source. The proposed SPDC process implies the entanglement between each pair in the collinear process that they are simultaneously obtained from the identical pump photon as follows:

5 $$|{\Phi }_{SPDC}\rangle =\left|{{\lambda }_{S}\rangle }_{1}\right|{{\lambda }_{I}\rangle }_{1}+\left|{{\lambda }_{S}\rangle }_{2}\right|{{\lambda }_{I}\rangle }_{2},$$

where $|{\Phi }_{SPDC}\rangle$ denotes the total SPDC state, $\left|{{\lambda }_{S}\rangle }_{i}\right|{{\lambda }_{I}\rangle }_{i}$ is the state of the type-0 signal-idler pair with each wavelength at ${\lambda }_{S}$ and ${\lambda }_{I}$, and $\left|{{\lambda }_{S}\rangle }_{j}\right|{{\lambda }_{I}\rangle }_{j}$ is of the corresponding simultaneous pair. Additionally, non-collinear processes allow the entangled state in the multi-dimension. This will be a novel scheme for multi-color entangled photon sources^20^ and serve for the broadband light source for QIS and quantum sensing. Therefore, implementation of this SPDC emitter as a quantum light source will open the way to measure quantum phenomena in the IR region as well as the broadband spectra for IR spectroscopy.

## Supplementary Information

Supplementary Information.
