# Supplementary material for: Broadband infrared light source by simultaneous parametric down-conversion
Source: Sci Rep. 2021 Sep 9;11:17986. doi: 10.1038/s41598-021-97531-w (PMC8429520; doi:10.1038/s41598-021-97531-w)
Supplement: Supplementary file 1 — Supplementary Information. [file 41598_2021_97531_MOESM1_ESM.docx]

**Supplementary Information**

**Broadband infrared light source by simultaneous parametric down-conversion**

**Masayuki Hojo and Koichiro Tanaka**

1. **Optimization of pump wavelength**

We describe the detail of the quasi-phase-matching (QPM) calculations. For $\Delta k=0$ in a non-collinear process, the equation (2) in the article is described as

$$\left( \frac{n\left( \lambda_{3} \right)}{\lambda_{3}} \right)^{2}=\left( \frac{n\left( \lambda_{1} \right)}{\lambda_{1}}-\frac{1}{\Lambda} \right)^{2}+\left( \frac{n\left( \lambda_{2} \right)}{\lambda_{2}} \right)^{2}-2\left( \frac{n\left( \lambda_{1} \right)}{\lambda_{1}}-\frac{1}{\Lambda} \right)\left( \frac{n\left( \lambda_{2} \right)}{\lambda_{2}} \right)\cos\theta_{2}. (S1)$$

Here $\theta_{2}$ indicates an angle of the idler against the pump direction, as shown in the Fig. 2(a) in the article. Figures S1(a) and S1(b) show the relation between the pump wavelength and the idler wavelength. The variation of colors corresponds to variable pump wavelengths. As shown in Figure S1(a), another idler is simultaneously phase-matched at more than 3 µm, which is determined by the pump wavelengths. Note that we calculated the relative periodicities$\Lambda_{rel}$as follows:

$$\Lambda_{rel}=\Lambda\left( \lambda_{2} \right)-\Lambda\left( 2\mu m \right),$$

where $\Lambda\left( \lambda_{2} \right)$ is the periodicity with the idler phase-matched at $\lambda_{2}$.

Considering the PPSLT transparent band 0.8-5.5 µm in the IR region, it is ideal that the idler pairs are covered at the range between 2-5 µm in the near-collinear process. One can see that around 750 nm pump is optimal as the blue curve shows in Figure S1(b), in which all components of the idlers are emitted at the angle within 5 degrees. We mentioned the case of 750 nm in Figures 3(a) and 3(b) in the article.

(b)

(a)


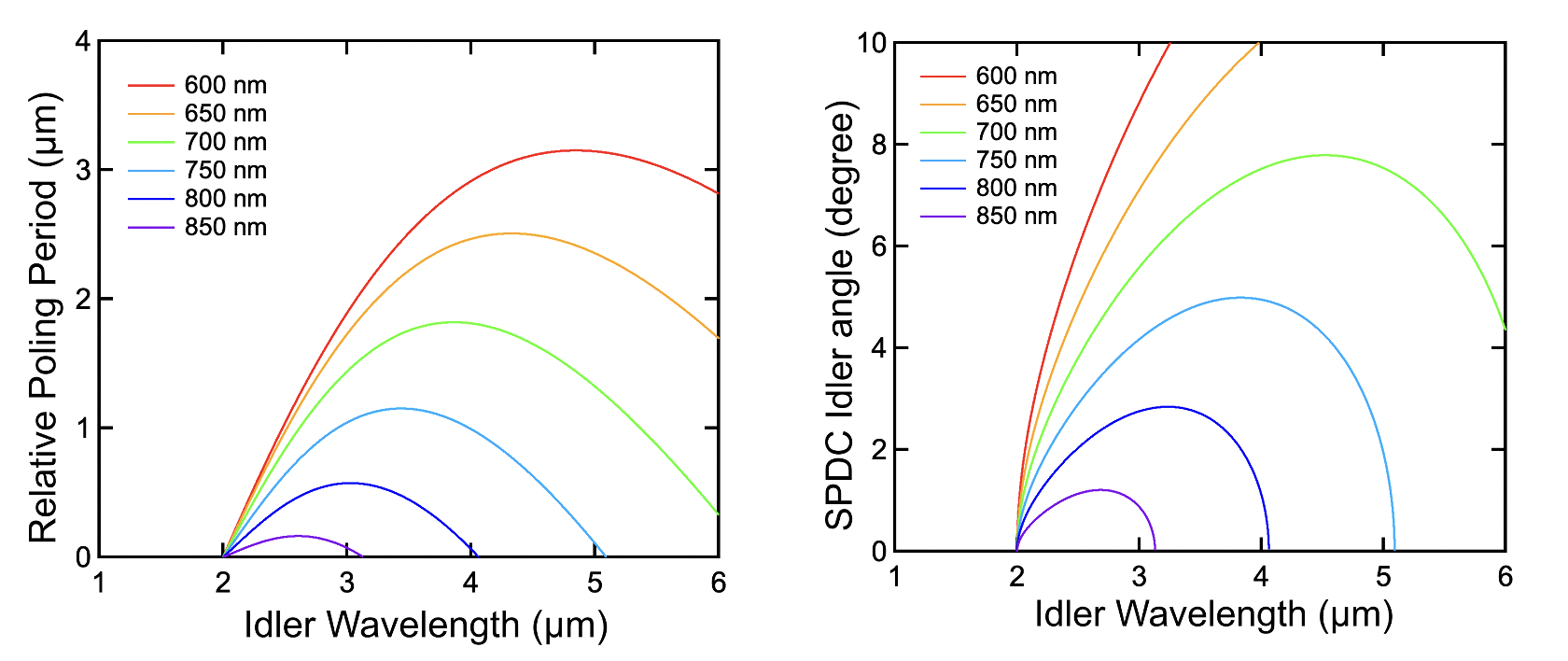


Fig. S1. (a) Comparison of QPM conditions for variable pumps. The vertical axis represents the relative period defined in equation (S2). The ideal condition is that two idlers are simultaneously phase-matched at 2 and 5 µm in the collinear configuration. (b) Relation between the idler output angle and wavelength. The periodicities are arranged so that one idler is emitted at 2µm in the collinear configuration

1. **Type-0 quasi-phase-matching condition for PPLN**

We investigated the type-0 quasi-phase-matching (QPM) condition (equation (2) in the article)

$$\vec{k_{1}}-\vec{k_{2}}-\vec{k_{3}}-\vec{G_{\Lambda}}=0 \left( S3 \right)$$

for PPLN using the temperature-dependent Sellmeier equation^1^. Figure S2 (a) shows the collinear QPM curve as a function of the SPDC wavelength, with $\lambda_{1}$ at 0.8 µm. For the simultaneous SPDC process, the period should be set in the range of 20.7-22.3 µm. At 21 µm period of PPLN, the angle dependence was calculated as shown in Fig. S2(b). One can find that the spectral window of the idler covers 2-5 µm within the emitting angle 0.1 rad. Thus, with the optimized pump for PPLN, broad spectral window can be also obtained in the near-collinear process as well PPSLT. Compared with the PPSLT, there is a little difference in terms of the optimized pump wavelength and the periodicity. However, both has a possibility to generate 2-5 µm IR light. We mentioned the case of PPLN in Figures 3(c) and 3(d) in the article.


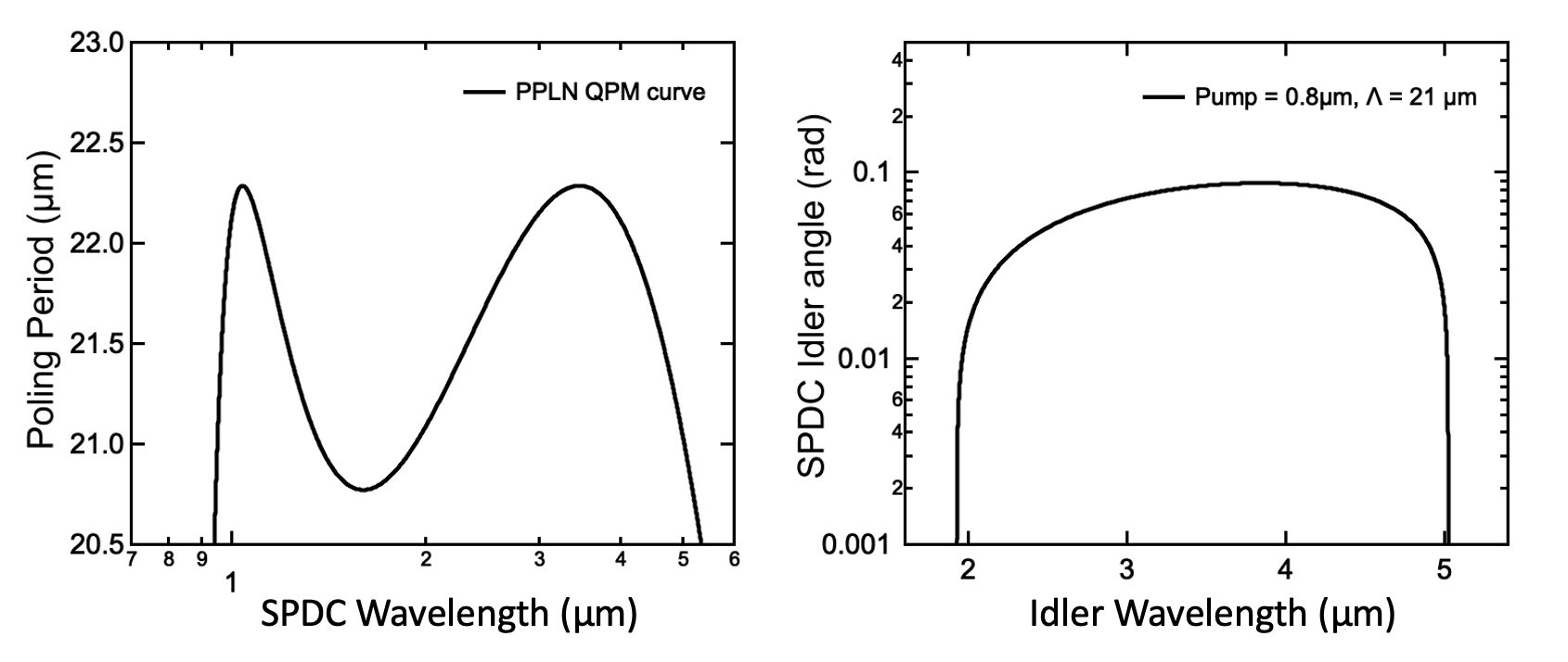


Fig. S2. (a) The QPM curve for the collinear SPDC process pumped at 0.8 µm. The bottom axis is in log-scale. (b) The angle dependence on the idler wavelengths. The left axis is in log scale.

(a)

(b)

1. **Estimation of the total power against the crystal length**

Here we show the theoretical calculation of the dependence of the total signal power on the crystal length *L* . The total power *P*(*L*) is calculated in the equation (5) as follows,

$$P\left( L \right)=\iint d\omega_{3}d\theta_{3}\frac{\omega_{3}^{3}\omega_{2}n_{3}^{2}L^{2}d^{2}P_{1}}{2\pi^{2}c^{5}\varepsilon_{0}n_{1}n_{2}}\frac{\sin\theta_{3}}{\cos^{3} \theta_{3}}\mathrm{Sin}c^{2}\left[ \frac{\Delta kL}{2} \right]. \left( S4 \right)$$

Figure S3 shows the relation of the power for the crystal length. The power is nearly proportional to *L*. This result is well agreed with the equation reported in the literature^2^. Therefore, we can obtain higher brightness when PPSLT has the length longer.


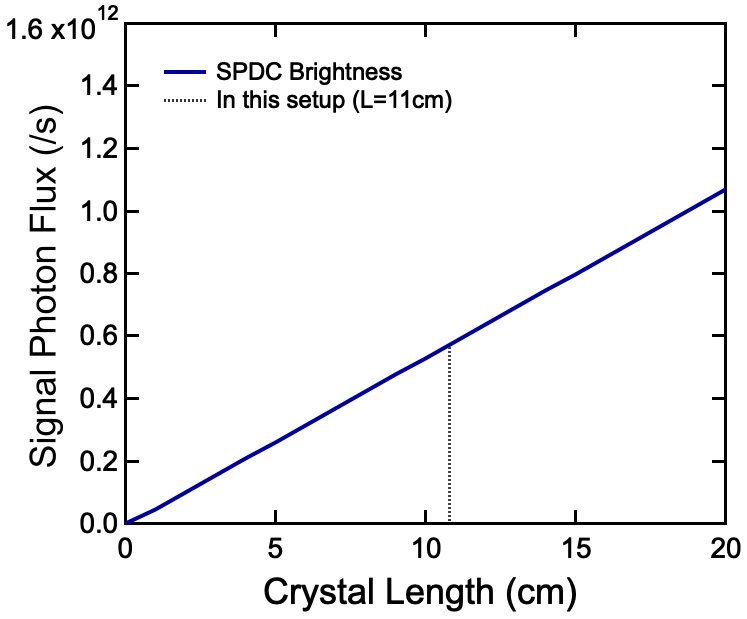


Fig. S3. Simulation of the power depending on the crystal length. Red marker indicated our experimental condition.

**Reference**

1. Gayer, O., Sacks, Z., Galun, E. & Arie, A. Temperature and wavelength dependent refractive index equations for MgO-doped congruent and stoichiometric LiNbO3. *Appl. Phys. B Lasers Opt.* **91**, 343–348 (2008).

2. Byer, R. L. & Harris, S. E. Power and bandwidth of spontaneous parametric emission. *Phys. Rev.* **168**, 1064–1068 (1968).
